# Evaluating Machine Learning–Based Automated Personalized Daily Step Goals Delivered Through a Mobile Phone App: Randomized Controlled Trial

**DOI:** 10.2196/mhealth.9117

**Published:** 2018-01-25

**Authors:** Mo Zhou, Yoshimi Fukuoka, Yonatan Mintz, Ken Goldberg, Philip Kaminsky, Elena Flowers, Anil Aswani

**Affiliations:** ^1^ Department of Industrial Engineering and Operations Research University of California Berkeley, CA United States; ^2^ Institute for Health & Aging School of Nursing University of California San Francisco, CA United States; ^3^ Department of Physiological Nursing School of Nursing University of California San Francisco, CA United States; ^4^ Department of Electrical Engineering and Computer Sciences University of California Berkeley, CA United States; ^5^ Institute for Human Genetics University of California San Francisco, CA United States

**Keywords:** physical activity, cell phone, fitness tracker, clinical trial

## Abstract

**Background:**

Growing evidence shows that fixed, nonpersonalized daily step goals can discourage individuals, resulting in unchanged or even reduced physical activity.

**Objective:**

The aim of this randomized controlled trial (RCT) was to evaluate the efficacy of an automated mobile phone–based personalized and adaptive goal-setting intervention using machine learning as compared with an active control with steady daily step goals of 10,000.

**Methods:**

In this 10-week RCT, 64 participants were recruited via email announcements and were required to attend an initial in-person session. The participants were randomized into either the intervention or active control group with a one-to-one ratio after a run-in period for data collection. A study-developed mobile phone app (which delivers daily step goals using push notifications and allows real-time physical activity monitoring) was installed on each participant’s mobile phone, and participants were asked to keep their phone in a pocket throughout the entire day. Through the app, the intervention group received fully automated adaptively personalized daily step goals, and the control group received constant step goals of 10,000 steps per day. Daily step count was objectively measured by the study-developed mobile phone app.

**Results:**

The mean (SD) age of participants was 41.1 (11.3) years, and 83% (53/64) of participants were female. The baseline demographics between the 2 groups were similar (*P*>.05). Participants in the intervention group (n=34) had a decrease in mean (SD) daily step count of 390 (490) steps between run-in and 10 weeks, compared with a decrease of 1350 (420) steps among control participants (n=30; *P*=.03). The net difference in daily steps between the groups was 960 steps (95% CI 90-1830 steps). Both groups had a decrease in daily step count between run-in and 10 weeks because interventions were also provided during run-in and no natural baseline was collected.

**Conclusions:**

The results showed the short-term efficacy of this intervention, which should be formally evaluated in a full-scale RCT with a longer follow-up period.

**Trial Registration:**

ClinicalTrials.gov: NCT02886871; https://clinicaltrials.gov/ct2/show/NCT02886871 (Archived by WebCite at http://www.webcitation.org/6wM1Be1Ng).

## Introduction

### Physical Inactivity

Physical inactivity is the fourth leading risk factor for mortality, causing an estimated 3.2 million deaths worldwide [1]. It is associated with cardiovascular disease, certain types of cancer, type 2 diabetes, and depression [2-5]. Moderate- to vigorous-intensity physical activity, such as brisk walking or running, has significant health benefits across all age groups. The 2008 National Physical Activity Guideline for Americans recommends at least either 150 min of moderate-intensity physical activity or 75 min a week of vigorous-intensity physical activity for adults [6]. However, approximately half of American adults, particularly women and minorities, do not meet this physical activity guideline [7,8].

### Mobile Health Interventions

Several lifestyle modification programs that promote physical activity have been demonstrated to be effective, but these programs are costly and labor-intensive because they require substantial in-person counseling [9-11]. To lower costs, researchers have conducted randomized controlled trials (RCTs) to investigate the feasibility of mobile health (mHealth) interventions (eg, mobile phone apps and digital pedometers) with reduced number of in-person counseling sessions [12-20]. Prior mHealth interventions implemented various goal-setting strategies to induce efforts, for example, to achieve and maintain 10,000 steps per day [21-26] or meet adaptively increasing step goals [14,27-29]. These studies demonstrated that mHealth interventions with goal setting can increase physical activity relative to baseline levels of activity.

### Goal Setting

Goal setting is known to be an important factor for facilitating behavior change [30-32], and effective goal setting requires self-monitoring to better enable attainment of goals and increase self-efficacy [14,30,31,33]. There are three considerations regarding goal setting: (1) self-set goals versus assigned goals versus participatory goals, (2) adaptive goals versus fixed goals, and (3) personalized goals versus nonpersonalized goals. Despite the fact that self-set goals are of higher personal importance, a review of the goal-setting literature [30] reveals that assigned goals are more effective compared with self-set goals because self-set goals require regular input from participants, which is more difficult to maintain. Furthermore, more recent RCTs reveal that increases in physical activity through mobile-only programs with fixed, nonpersonalized physical activity goals are often substantially lower than increases in physical activity through programs that include adaptive goals [28,29,34,35] or personalized goal setting provided during in-person counseling [13,34,36-39]. For instance, one study [29] found that setting adaptive step goals resulted in an increase of 1130 more steps between baseline and 6 months, compared with setting fixed step goals of 10,000. Studies suspect that assigning nonpersonalized, fixed goals to all participants can lead to unrealistically high goals for some participants and unchallenging goals for other participants, which reduces goal-setting effectiveness [37,40]. Therefore, assigning adaptively personalized goals can be a favorable alternative to better induce efforts and increase physical activity [40-42]. Personalized, adaptive goal setting allows changing goals over time based on prior individual behavior. For example, future daily step goals can be assigned based on step totals from the previous days to ensure that the goals are challenging yet realistic for each individual. Two trials [28,29] used the same approach by combining financial incentives for meeting goals with an adaptive approach that set goals for the next day to be the 60th percentile of the steps taken in the past 10 days. Although this simple adaptive goal algorithm was modestly effective [28], a computer simulation study [43] for a weight loss intervention involving physical activity goal-setting and in-person counseling sessions found that a more sophisticated algorithm using statistics and machine learning to set goals by learning participants’ responsiveness to goals could provide greater effectiveness (as compared with simple rules such as goal setting using a fixed percentile of steps taken in the past few days) in encouraging individuals to increase their physical activity and lose weight. In particular, the simulation showed that (when each participant received four counseling sessions) the more sophisticated machine learning algorithm would encourage almost half of the participants to have 5% or more body weight loss, whereas the use of goal setting using a fixed percentile of steps taken in the past few days would encourage only about one-quarter of the participants to have 5% or more body weight loss. Furthermore, previous studies have found that financial incentives may be effective during the intervention period, but in the maintenance period, participants are more likely to not adhere when no financial incentive is given [44-46].

### Study Purpose

The purpose of our study was to test a sophisticated algorithm for personalized, adaptive goal setting that uses statistics and machine learning [43,47], and specifically to examine its efficacy in a fully automated mobile phone–based intervention with no in-person contact or counseling sessions during the trial. It is important to note that goal setting is only one component of a behavior change intervention, and our study is designed to isolate the impact of goal setting from other components to evaluate the efficacy of goal setting alone. We developed an automated mobile phone–based iPhone operating system (iOS, Apple Inc) app named CalFit, which sets personalized, adaptive step goals using the behavioral analytics algorithm (BAA) [43,47], and conducted an RCT (called Cal Fitness) using this mobile phone app in the United States. To our best knowledge, this is the first app implementing BAA. BAA first uses machine learning to construct a predictive quantitative model for each participant based on the historical step and goal data, and then, it uses the estimated model to generate challenging yet realistic step goals in an adaptive fashion by choosing step goals that, based on the estimated model, would maximize future physical activity. The primary aim of this RCT was to evaluate the efficacy of the automated mobile phone–based personalized, adaptive goal-setting intervention as compared with the active control with nonpersonalized, steady daily step goals of 10,000. The main outcome measure was the relative change in objectively measured daily steps between the run-in period and 10 weeks. Secondary outcome measures included the following: step goal attainment (ie, fraction of step goals achieved by each participant), weight and height, self-reported sociodemographic information, self-reported medical history, Barriers to Being Active Quiz [48], and the short version of the international physical activity questionnaire [49]. We collected these survey results to investigate if the goal-setting component alone is capable of changing participants’ survey responses before and after the study.

## Methods

### Study Design

The Cal Fitness study was a 10-week RCT with 2 groups: (1) the intervention group received automated personalized daily step goals, and (2) the control group received fixed daily step goals of 10,000 steps per day. The study was approved by the Committee for Protection of Human Subjects at the University of California, Berkeley (UCB; institutional review board number 2016-03-8609), in July 2016 and was registered with the clinicaltrials.gov (NCT02886871) in August 2016. All participants provided written informed consent before study enrollment. This RCT was conducted in 2016 and analyzed in 2017.

### Participant Recruitment

A total of 64 adult staff employees of UCB were recruited via email announcements. Recruitment commenced in August 2016 and ended in September 2016. The study ended in December 2016 to allow a 10-week period to all participants. Potential participants were contacted through email and then directed to a Web-based screening survey to assess eligibility. Those participants who met all the inclusion criteria were then contacted by trained study personnel via email to arrange an in-person session. Ineligible participants were informed by email to advise them that they are ineligible, and corresponding data were deleted.

The inclusion and exclusion criteria for the Cal Fitness study are given in Textbox 1.

### Study Procedure

Eligible participants were asked to attend two 15-min in-person sessions (initial and 10-week post intervention visits) at UCB. The first in-person session occurred in September 2016, and the second session occurred in December 2016. During the first in-person session, a trained research staff member installed the CalFit app on participants’ phones and advised the participants to keep the phone in their pocket or purse for the following 10-week period. A trained research staff member measured height (cm) and weight (kg) in both the sessions using a Seca 700 Physician’s Balance Beam Scale with Height Rod, and body mass index (BMI) was also calculated. Participants were then instructed to complete the sociodemographic survey, the medical history survey, the Barriers to Being Active Quiz [37], and the short version of the international physical activity questionnaire [38]. During the second in-person session, a trained research staff removed the CalFit app from participants’ phones. Participants were then instructed to complete the Barriers to Being Active Quiz [37] and the short version of the international physical activity questionnaire [38]. Participants received a US $50 Amazon gift certificate at completion if they completed all study requirements.

**Inclusion and exclusion criteria for the Cal Fitness study.**

Inclusion criteria

- Staff member of University of California, Berkeley
- Intent to become physically active in the next 10 weeks, which was evaluated by asking potential participants if they wanted to increase their physical activity beyond their self-assessed current level
- Own an iPhone 5s (or a newer model)
- Willing to keep the iPhone in pockets during the day
- Willing to install and use the study app (which requires Internet connection) every day for 10 weeks
- Ability to speak and read English

Exclusion criteria

- Known medical conditions or physical problems that require special attention in an exercise program
- Planning an international trip during the next 3 months, which could interfere with daily server uploads of mobile phone data
- Pregnant or gave birth during the past 6 months
- Severe hearing or speech problem
- History of an eating disorder
- Current substance abuse
- Current participation in lifestyle modification programs or research studies that may confound study results
- History of bariatric surgery or plans for bariatric surgery in the next 12 months

### CalFit iOS App

**Figure 1:**
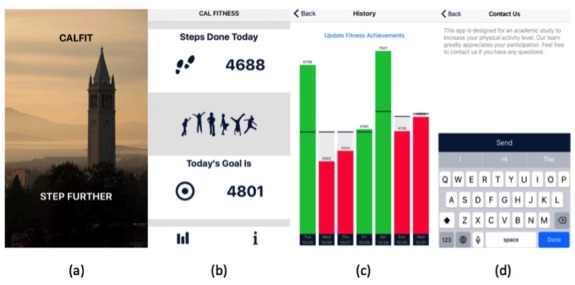
The CalFit app interface. (a) The landing page; (b) The homepage showing the steps done today and today’s goal; (c) The "History" tab showing the performance of the past week. The black bar is the goal, and the bars are green for achieved goals and red for unachieved goals; (d) The "Contact Us" tab where participants can easily send messages to the study team.

Our research team developed the CalFit app (iOS version, Apple Inc), which was designed to increase physical activity by allowing participants to track their daily step goals and to compare their step counts with their daily step goals in the past. Figure 1 shows the interface of the CalFit app. After participants open the app, they see the landing page and then the home page. On the home page, *number of steps completed today* and *today’s step goal* are shown. Participants can click on two icons at the bottom of the home page. If they click on the left icon, the history page is displayed. The history page allows participants to track their performance over the past week by showing their daily steps and daily goals on a color-coded bar graph. The green bar indicates the accomplishment of achieving the step goal on the corresponding day, and the red bar indicates failure to achieve the step goal on the corresponding day. When participants click on the right icon, they reach the contact page that allows them to send messages to the research team. The built-in health chip in the iPhone collects the step data, and the accuracy of step counts collected by the iPhone health chip has been validated in a number of studies to have comparable accuracy to an ActiGraph [50-55]. One of these studies [51] conducted a large number of experiments and concluded that iPhones are accurate for tracking step counts, with a relative difference in the mean step count of −6.7% to 6.2% compared with direct observation. Another study [55] compared iPhone pedometer measurements with measurements from wearable devices in a free-living setting and concluded “measurements of number of steps and distance were excellent and could provide reliable judgment on the individuals’ activity amount.” Our app first saves the step and goal data locally on the phone and then syncs with the server every 10 min when the phone is active. The push notification for the app is also activated, and the standard iOS push notification is used. The push notification is visible in the landing page and in the recent notifications tab on the phone.

### Run-In Period and Randomization

A total of 64 eligible participants started a 1-week run-in period after completing the initial in-person session. The purpose of the run-in period was to collect run-in daily steps, and assess if the participant was able to comply with the requirements needed to regularly use the CalFit app. During the run-in period, all participants in the control and the intervention groups received the identical set of daily step goals for day 1 to day 7 as 3000, 3500, 4000, 4500, 5000, 5500, and 6000 steps, respectively. The BAA algorithm was not used to compute step goals for participants in the intervention group during the run-in period. Dynamically increasing step goals were used in the run-in period to engage participants in using the app regularly. In addition, all participants received a push notification at 8 AM that provided today’s step goal, and if the participant accomplished the goal before 8 PM, then another push notification was sent to congratulate the participant on reaching their step goal for the day. The identical goals between the 2 groups during the run-in period is used to establish a reference level of initial physical activity, which we used in our statistical analyses to compare the difference in daily steps between run-in and 10 weeks for the 2 groups. Data collected during the run-in period were used by the BAA algorithm to compute step goals for the intervention period. This is a valid approach because run-in data were indicative of the preference of different participants. All 64 participants were randomized to one of the 2 groups with a one-to-one ratio by a computer-based random number generator using the simple randomization approach. A one-to-one ratio means that each participant had a 50% probability of being assigned to one of the 2 groups, and the number of participants in each group may differ due to chance. The randomization to groups was implemented by the CalFit app after the run-in period, and the participants were aware of the 2 groups.

### Control

After the 1-week run-in period, participants in the control group were provided with constant daily step goals that were set to 10,000 steps per day through the CalFit app. Participants received a push notification at 8 AM every day that provided that day’s step goal (10,000 steps), and if the participant achieved the goal before 8 PM, then another push notification was sent to congratulate the participant on reaching their step goal (of 10,000 steps) for the day.

### Intervention

After the 1-week run-in period, participants in the intervention group received adaptively personalized step goals through the CalFit app. The daily step goals were computed using the BAA [43,47] on the complete history (past steps and goals) of the user. The BAA algorithm was applied every week to reduce variance in future steps and goals. Participants received a push notification at 8 AM every day that provided today’s step goal, and if the participant accomplished the goal before 8 PM, then another push notification was sent to congratulate the participant on reaching their step goal for the day.

A rigorous mathematical formulation of the BAA algorithm that we used is provided in 2 studies [43,47]. This algorithm uses statistics and machine learning to adaptively compute personalized step goals that are predicted to maximize future physical activity for each participant based on all the past steps’ data and goals of each participant. The BAA algorithm is applied to each participant individually, and it consists of two main steps. The first step is to use all of the participant’s data to construct a quantitative model that predicts how many steps the participant will take in the future, given a prescribed set of step goals, and an important aspect of the model is a component that describes how achieving goals in the present can increase the likelihood of achieving goals in the future. The second step is to use this quantitative model to select a sequence of step goals that maximizes the predicted future number of steps. To make the process of updating step goals adaptive, the BAA algorithm is applied each week (using all the users’ past data) to generate step goals for the coming week; moreover, the step goals computed by the BAA algorithm for the coming week are not constant, but increase or decrease based on the model prediction. A computer simulation study [43] found that applying the algorithm weekly is as effective as applying the algorithm daily (because steps can vary significantly on a day-to-day basis), and so we applied the algorithm weekly. More details about the BAA algorithm are provided in 2 studies [43,47].

### Outcome Measures

The primary outcome of the study was the relative change in daily steps from run-in to the 10-week follow-up, measured objectively by the participants’ iPhones. The daily step values were compared in the manner described in the statistical analysis section. Step count data were stored in a database on a private computer server at UCB. Data were automatically synced with the iPhone once every 10 min during the study. At the 10-week in-person session, complete step data were downloaded from the iPhone to store step count data that were unable to be transmitted. Data were unable to be transmitted if the app was turned off or no Internet connection was available. Other measures included weight and height, self-reported sociodemographic information, self-reported medical history, Barriers to Being Active Quiz [48] (which consists of 21 questions on a 10-point Likert scale on 7 subareas: lack of time, social influence, lack of energy, lack of willpower, fear of injury, lack of skill, and lack of resources), and the short version of the international physical activity questionnaire [49].

### Statistical Analysis

Assuming an expected loss to follow-up of 10%, a target sample size of 30 participants per group was selected to give 80% power to detect between-group difference of 1500 steps with a pooled standard deviation (SD) of 2000 using a two-sided test and an alpha of .05. Differences between groups in run-in and 10 weeks were assessed using Student *t* test. The statistical analysis of the primary outcome of daily steps was performed using a linear mixed-effects model (LMM) with piecewise linear growth curve [56-58] with random effects for each individual of random slope and random intercept, and fixed effects of time, treatment group, and interaction term of time and treatment group. Our statistical analysis of the secondary outcome of step goal attainment (ie, fraction of step goals achieved by each participant) was performed by a similar LMM but with an additional specification of a binary response variable (ie, goal is either attained or not attained by an individual on a particular day). Means with 95% confidence intervals were obtained from the LMM. Sensitivity analysis was performed to obtain adjusted estimates of the effect of the treatment with the missing data on primary outcome, evaluated at *P*<.05. The primary cause of missing step data was failure to turn on the app. LMM implicitly imputes missing data by interpolation and is a common approach to deal with missing data in physical activity interventions [56-60]. (We did not use the common imputation method of “last observation carried forward” because it would increase bias in this context and lead to potentially false conclusions by inflating step counts at 10 weeks.) For accurate comparison between the control and the intervention groups, the weekly average steps in run-in were adjusted by adding the coefficient corresponding to each group (ie, control or intervention) computed by the LMM model. In addition, weekly moving average steps were computed by taking the average of each moving window with length 7, to reduce noise for better visualization.

To quantify app use for the intervention, a participant was categorized as a nonfrequent app user if the app was not used for a consecutive period of 7 days. By this criterion, 17 participants out of 34 in the intervention group and 16 participants out of 30 in the control group were frequent app users. Per-protocol analysis was performed on the 33 frequent app users, and intention-to-treat analysis was performed on all 64 subjects. Although the power for the per-protocol analysis will be low, the reason for conducting this analysis is that we want to investigate the impact of the CalFit app on an active subgroup, which could be more representative for its true performance if adopted in other full interventions that include additional components of a behavior change intervention. Intention-to-treat analysis was performed for the primary and secondary outcomes, and per-protocol analysis was performed only for the primary outcome. Missing survey response data resulting from lost to follow-up was imputed by the latest available survey response of the subject. The statistical analysis was performed in MATLAB (MathWorks, Massachusetts, USA) version 9.0 [61] and R (R Core Team, Vienna, Austria) version 1.0.136 [62] in the year 2017.

## Results

### Recruitment Results

**Figure 2:**
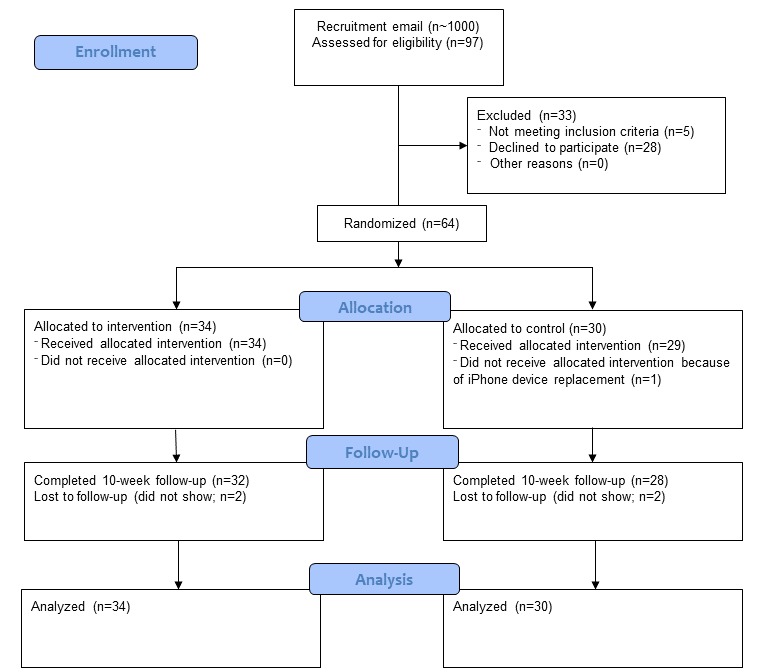
Screening, randomization, and assessments of study participants.

As shown in Figure 2, 97 potential participants were screened for eligibility by an online form, and 64 completed the initial in-person session.

### Baseline Characteristics

Table 1 shows the baseline characteristics of the participants. A total of 34 participants were randomly assigned to the intervention group, and 30 participants were randomly assigned to the control group. All participants were included in the analysis based on the original assigned groups. Overall mean age was 41.1 (SD 11.3) years, and 83% (53/64) participants were female. In addition, 55% (35/64) of the participants self-identified as a member of a racial minority group. The baseline mean weight of participants was 77.2 kg (SD 18.7 kg) and the mean BMI was 27.3 kg/m^2^(SD 6.1 kg/m^2^). The mean height and weight for male and female participants were 177.5 cm and 82.6 kg and 165.9 cm and 76.1 kg, respectively. Furthermore, 20% of the participants reported at least one medical condition (ie, high blood pressure, type 2 diabetes, type 1 diabetes, coronary heart disease, or hypercholesterolemia). No baseline characteristics differed between the control and intervention groups. The run-in mean daily steps in the control and intervention groups were similar (7427 steps vs 7237 steps, respectively; *P*=.79) and are in line with baseline steps in other similar studies [14,42,63,64]. As shown in Multimedia Appendix 1, the self-reported survey results did not differ considerably between the 2 groups except for the lack of resources, which is a subscale of the Barriers to Being Active measure. The intervention group had a significantly higher rating of lack of resources than the control group (*P*=.03). We suspect this significant difference for lack of resources occurred due to chance.

**Table 1:** Baseline characteristics between the control and intervention groups.

Baseline characteristics			All participants (N=64)	Control (N=30)	Intervention (N=34)	*P*
Run-in daily average steps, mean (SD)			7326 (2907)	7427 (2398)	7237 (3326)	.79
Age, years, mean (SD)			41.1 (11.3)	40.5 (10.5)	41.6 (12.2)	.72
Weight, kg, mean (SD)			77.2 (18.7)	77.8 (21.3)	77.0 (17.1)	.87
BMI^a^, kg/m^2^, mean (SD)			27.3 (6.1)	27.1 (6.7)	27.4 (5.8)	.82
**Gender, n (%)**						.82
	Male		11 (17)	6 (20)	5 (15)	
	Female		53 (83)	24 (80)	29 (85)	
**Ethnicity, n (%)**						.86
	Asian		13 (20)	7 (23)	6 (18)	
	Black or African American		8 (13)	3 (10)	5 (15)	
	Hispanic or Latino		9 (14)	5 (17)	4 (12)	
	White or non-Hispanic		29 (45)	13 (43)	16 (47)	
	Other		5 (8)	2 (7)	3 (9)	
**Marital status, n (%)**						.20
	Currently married or cohabitating		36 (56)	15 (50)	21 (62)	
	Never married		21 (33)	13 (43)	8 (24)	
	Divorced or widowed		7 (11)	2 (7)	5 (15)	
**Education, n (%)**						.30
	Completed some college		5 (8)	1 (3)	4 (12)	
	Completed college (4 years)		28 (44)	12 (40)	16 (47)	
	Completed graduate school		31 (48)	17 (57)	14 (41)	
**Work hour (per week), n (%)**						.17
	1-20 hours		3 (5)	3 (10)	0 (0)	
	21-40 hours		16 (25)	7 (23)	9 (27)	
	>40 hours		45 (70)	20 (67)	25 (74)	
**Own a dog, n (%)**						.99
	Yes		16 (25)	8 (27)	8 (24)	
	No		48 (75)	22 (73)	26 (77)	
**Transportation to work, n (%)**						.49
	Car		28 (44)	10 (33)	18 (53)	
	Public transportation		25 (39)	14 (47)	11 (32)	
	Walk		4 (6)	2 (7)	2 (6)	
	Bicycle		6 (9)	3 (10)	3 (9)	
	Other		1 (2)	1 (3)	0 (0)	
**Gym membership, n (%)**						.45
	Yes		32 (50)	13 (43)	19 (56)	
	No		32 (50)	17 (57)	15 (44)	
**Self-reported medical history, n (%)**						
	**High blood pressure**					.88
		Yes	5 (8)	3 (10)	2 (6)	
		No	59 (92)	27 (90)	32 (94)	
	**Type 2 diabetes**					.43
		Yes	5 (8)	1 (3)	4 (12)	
		No	59 (92)	29 (97)	30 (88)	
	**Type 1 diabetes**					.62
		Yes	0 (0)	0 (0)	0 (0)	
		No	64 (100)	30 (100)	34 (100)	
	**Coronary heart disease**					.62
		Yes	0 (0)	0 (0)	0 (0)	
		No	64 (100)	30 (100)	34 (100)	
	**Hypercholesterolemia**					.83
		Yes	7 (11)	4 (13)	3 (9)	
		No	53 (83)	24 (80)	29 (85)	
		Unknown	4 (6)	2 (7)	2 (6)	

^a^BMI: body mass index

### Efficacy of Intervention

#### Main Analysis

**Figure 3:**
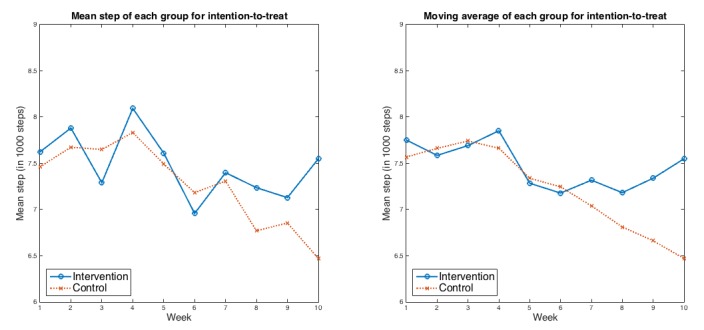
Weekly average and moving average steps for the 2 groups over the course of the study for intention-to-treat analysis after run-in adjustment. Left panel: mean weekly steps for intention-to-treat; Right panel: weekly moving average for intention-to-treat.

Intention-to-treat analyses indicated that the intervention group had a decrease in mean (SD) daily step count of 390 (SD 490) steps between run-in and 10 weeks compared with a decrease of 1350 (SD 420) steps among controls (*P*=.03). The net difference in daily steps between the groups was 960 steps (95% CI 90-1830 steps). Table 2 shows the run-in adjusted objectively measured raw average weekly steps for both the groups without missing data imputation. Figure 3 shows the run-in adjusted weekly average steps and moving average steps for intention-to-treat.

**Figure 4:**
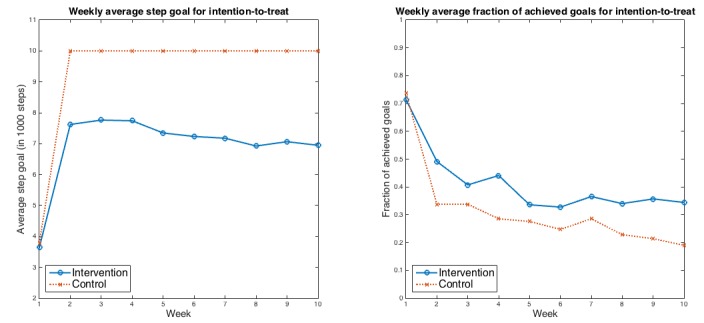
Weekly average step goals and average fraction of goals achieved for the 2 groups for intention-to-treat analysis. Left panel: weekly average step goals for intention-to-treat; Right panel: weekly average fraction of achieved goals for intention-to-treat.

The average step goals for the first week are the same for both the control and the intervention groups because both received the same goals during the first week. Table 3 gives the fraction of achieved step goals for the 2 groups. Intention-to-treat analysis indicated that the intervention group had a decrease in mean fraction of achieved step goals of 0.34 (SD 0.05) between run-in and 10 weeks compared with a decrease of 0.49 (SD 0.04) among controls (*P*=.003). The net difference in fraction of achieved step goals between the groups was 0.15 (95% CI 0.02-0.25). Figure 4 details the intention-to-treat weekly average step goals and the fraction of achieved step goals for the 2 groups.

#### Sensitivity Analysis

**Figure 5:**
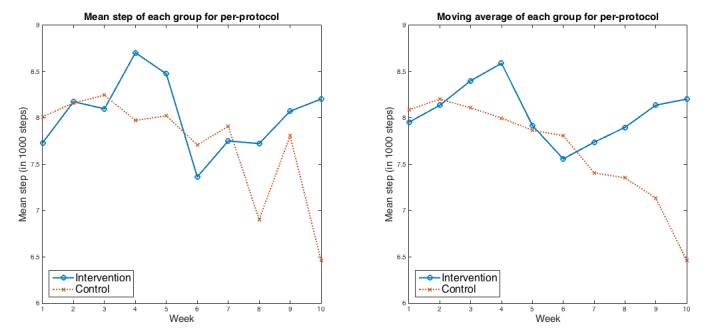
Weekly average and moving average steps for the 2 groups over the course of the study for per-protocol analysis after run-in adjustment. Left panel: mean weekly steps for per-protocol; Right panel: weekly moving average for per-protocol.

Per-protocol analysis (among the 33 frequent app users: 16 in control and 17 in intervention groups) indicated that the intervention group had a decrease in mean (SD) daily step count of 0 (SD 420) steps between run-in and 10 weeks, whereas the control group had a decrease of 1500 (SD 550) steps (*P*=.03). The net difference in daily steps between the groups was 1500 steps (95% CI 130-2900 steps). Figure 5 shows the run-in adjusted weekly average steps and moving average steps for per-protocol.

**Figure 6:**
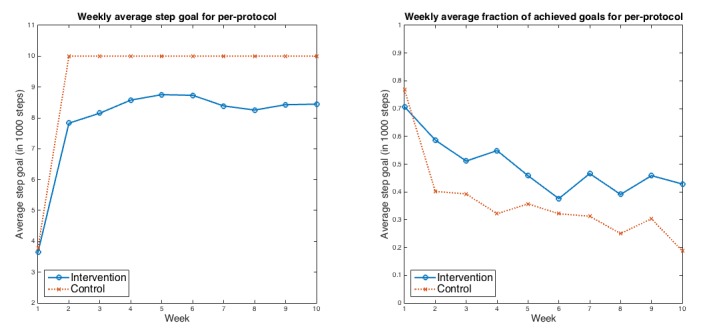
Weekly average step goals and average fraction of goals achieved for the 2 groups for per-protocol analysis. Left panel: weekly average step goals for per-protocol; Right panel: weekly average fraction of achieved goals for per-protocol.

Per-protocol analysis also indicated that the intervention group had a decrease in mean (SD) fraction of achieved step goals of 0.27 (SD 0.08) between run-in and 10 weeks compared with a decrease of 0.46 (SD 0.06) among controls (*P*=.02). The net difference in fraction of achieved step goals between the groups was 0.19 (95% CI 0.02-0.38). Figure 6 details the per-protocol weekly average step goal and the fraction of achieved step goals for the 2 groups.

#### Other Analysis

No significant difference (Multimedia Appendix 1) in self-reported physical activity scores and Barriers to Being Active was noted within the 2 groups over time, and no significant difference between the 2 groups was observed at run-in or 10 weeks.

### Fidelity of In-Person Sessions

The number of participants in the 2 groups who failed to complete the second in-person session for follow-up did not differ (*P*=.90): 6.6% (n=2) in the control group and 5.9% (n=2) in the intervention group (Figure 2). Their data were included in the analysis.

**Table 2:** Run-in adjusted objectively recorded (using iPhone) physical activity.

Week	Mean number of steps	
	Control (N=30)	Intervention (N=34)
Run-in	7462	7623
Week 2	7674	7882
Week 3	7650	7290
Week 4	7834	8094
Week 5	7494	7611
Week 6	7183	6958
Week 7	7308	7399
Week 8	6770	7237
Week 9	6855	7129
Week 10	6471	7549

**Table 3:** Fraction of achieved daily step goals in weeks.

Week	Control (N=30)	Intervention (N=34)
Week 1 (run-in)	0.74	0.71
Week 2	0.34	0.49
Week 3	0.34	0.41
Week 4	0.29	0.44
Week 5	0.28	0.34
Week 6	0.25	0.33
Week 7	0.29	0.37
Week 8	0.23	0.34
Week 9	0.21	0.36
Week 10	0.19	0.34

## Discussion

### Principal Findings

This study evaluated the efficacy of a mobile phone–based physical activity intervention that provided adaptively personalized daily step goals. The intervention led to a statistically significant difference of 960 more daily steps in the intervention group compared with the control group over 10 weeks, in line with similar studies [28,29]. Although both groups had reduced daily steps at 10 weeks as compared with run-in, we speculate this was caused by run-in step counts being higher than the natural baseline. We believe this inverse relationship was a result of participants receiving step goals and monitoring step count through the CalFit app or the built-in iPhone Health app during the run-in period. This is supported by the observations that during the run-in period, all participants received daily step goals of 3000, 3500, 4000, 4500, 5000, 5500, and 6000 steps and initially over responded to these goals, and that the trends in daily steps between the control and intervention groups began to diverge in the 6th week of the study when enthusiasm of study participants wore out. Thus, later in the study, the personalized daily step goals seemed to be more effective in engaging participants and maintaining daily step counts compared with constant step goals.

The health literature has identified that setting goals is effective in lifestyle modification and physical activity promotion [9,14,36,65]. One analysis found that the importance of goal attainment and self-efficacy are the two main factors that contribute to goal commitment [32]. More recent studies [66-68] showed that individuals with higher self-efficacy are more likely to achieve activity goals and that failing to achieve activity goals reduces individuals’ self-efficacy. Therefore, activity goals need to be set with care. Past studies [69-71] and most persuasive technologies [34,72] either adopted a steady goal of 10,000 steps or allowed self-set goals. To our knowledge, this is the first study to use machine learning to automatically set adaptively personalized step goals and deliver the step goals using a mobile phone technology. The RCT outcomes show that adaptively personalized goals were important in promoting physical activity relative to constant step goals. The adaptive step goals were set to be challenging yet attainable; thus, the average step goals for the intervention group were lower than the average step goals for the control group. As the adaptive step goals were designed to be challenging, the goal achieving percentage for the intervention group was not 100%. Instead, we observed the goal achieving percentage for the intervention group was 30%-40%, which was 15% more compared with the goal achieving percentage for the control group. Being able to achieve more daily step goals can enhance participants’ self-efficacy, which further promotes physical activity in the days to follow [32,73-75]. The significantly higher (but not too high) rate of achieving step goals and significantly more steps of the participants in the intervention group demonstrate that the BAA algorithm computed adaptively personalized step goals that were capable of being both challenging and manageable for participants, and these goals effectively promoted physical activity.

Nonadherence is another challenge in mobile phone–based lifestyle modification programs. As a result, many past mHealth interventions involve regular in-person counseling sessions besides the mobile intervention to motivate adherence [9,14,76]. However, in-person counseling sessions are costly and put a burden on both the participants and the research staff [77-79]. Our study was intentionally designed to have only two in-person sessions (each of 15 min) at run-in and at 10 weeks to better simulate the environment of a completely mobile phone–based physical activity intervention. Note that the two in-person sessions in this study were necessary in-person contacts for the purpose of assessment in the study; they are different from in-person counseling sessions that serve as an essential part of an actual intervention. Despite the absence of coaching sessions, the percentage of frequent users observed over 10 weeks in our study was better than that reported in similar trials [71,80,81]. Our results indicate that a mobile phone–based intervention without coaching sessions is still effective in promoting physical activity. In-person contact and coaching sessions are therefore not necessary requirements for effective physical activity interventions, and there is potential to replace those contacts with better-designed physical activity apps.

An additional advantage of this study is that it only relied on one device for both data collection and intervention delivery. Similar studies either used a pedometer or accelerometer besides the mobile phone or requested regular data inputs from the participants, requiring greater efforts on the participant side, which was shown to be burdensome and could lead to declining use of the app [82]. In this study, step data were objectively measured by the iPhone, and participants were only requested to carry their mobile phones with them (in their pocket or their purse). No other manual data entry was needed on the participant side. Moreover, the CalFit app is designed in a flexible way that is compatible with other data collection devices, such as wearable step trackers, as long as the step data can be synced with the iPhone.

In addition to objectively measured outcomes, it is of interest to investigate if self-reported survey results differ between this study and full behavioral interventions (with many behavior change components). Barriers to Being Active quiz and the international physical activity questionnaire are popular surveys that have been widely adopted [14,33,81,83-85]. Researchers found that there exist significant differences in survey responses before and after a full behavioral intervention [14,33,86,87]. However, we failed to observe such difference. We suspect that goal setting alone may not be strong enough to change participants’ opinion on self-reported surveys and that other behavior change components are required (eg, coaching sessions).

This study tested one single component of behavior change (ie, goal setting), and the purpose of this design was to isolate the impact of goal setting from other behavior change components. Beyond goal setting, there are many other components of behavior change that can be beneficial for fitness apps. For instance, customized messages and social interactions have the potential to further improve the efficacy of fitness apps. This study is not designed to be a stand-alone intervention but rather to provide evidence on the efficacy of evaluating a single design component to motivate future evaluations on other design components. We believe there is great potential for better-designed fitness apps that can contribute to more effective physical activity intervention.

### Limitations

The first limitation of this study is the relatively small sample size, which only contained UCB adult staff workers with a dominant proportion of females (83% [53/64]). The results may not generalize to the general public. The relatively high education level of the participants may also limit the generalizability. In addition, the CalFit app was only available on the iOS platform, which could bias results. Second, the daily steps assessment during the run-in period was not able to establish a natural baseline. Therefore, our trial could only determine the relative (to the control) benefit of the intervention, but could not determine the absolute (compared with the natural baseline) benefit of the intervention. Blocked display of step counts with no step goal during the run-in period may provide additional insights to the natural baseline. Third, the iPhone was not able to collect data when it was being turned off or was not with the participant, and it was not able to distinguish the carrying method (purse vs pocket). However, the chance of the above happening was the same for the control and the intervention groups because of randomization; so, these factors do not impact the relative step differences between the 2 groups. Fourth, this study did not assess the underlying behavior skills (self-efficacy, goal setting, etc) that may impact individual’s response to interventions. Finally, the study was conducted for 10 weeks, which is a relatively short time. Studies that span a longer period are needed to evaluate the long-term effect of such personalized step goal-setting intervention delivered via mobile phones.

### Conclusions

Our RCT indicates that mobile phone–delivered adaptively personalized step goals are promising in promoting physical activity. The intervention led to a statistically significant difference of 960 more daily steps in the intervention group compared with the control group over 10 weeks. The higher (but not too high) percentage of goal achievement in the intervention group confirms that the adaptively personalized step goals computed by the BAA algorithm used in this trial are capable of creating challenging yet attainable goals. The significant step difference between the 2 groups suggests that a mobile phone–based physical activity intervention with reduced in-person sessions is feasible. The results obtained in this study can guide the design of future mobile phone–based physical activity interventions.

## Appendix

Multimedia Appendix 1 Self-reported physical activity scores and barriers to being active of the two groups pre and post the study.

Multimedia Appendix 2 CONSORT-EHEALTH checklist (V 1.6.1).

## Abbreviations

BAA: behavioral analytics algorithm
BMI: body mass index
iOS: iPhone operating system
LMM: linear mixed-effects model
mHealth: mobile health
PCARI: Philippine-California Advanced Research Institutes
RCT: randomized controlled trial
UCB: University of California, Berkeley
