# Supplementary material for: Evaluating Machine Learning–Based Automated Personalized Daily Step Goals Delivered Through a Mobile Phone App: Randomized Controlled Trial
Source: JMIR Mhealth Uhealth. 2018 Jan 25;6(1):e28. doi: 10.2196/mhealth.9117 (PMC5806006; doi:10.2196/mhealth.9117)
Supplement: Multimedia Appendix 1 [file mhealth_v6i1e28_app1.pdf]

Appendix 1

Table A1. Self-reported physical activity scores and barriers to being active of the two groups pre and post the study.

Variable	Range of possible scores	Control (M±SD)	Intervention (M±SD)	*P*	Overall *P*
**IPAQ Questionnaire**					
**Satisfied with physical activity level in the past week**	0-10 (10 being most, 0 being least)				0.69
**Run-in**		5.0±2.3	5.1±2.6	0.92	
**10-week**		5.8±2.7	5.3±2.6	0.45	
**Regular exercise routine**	0-10				0.17
**Run-in**		5.4±2.9	4.7±3.5	0.38	
**10-week**		5.9±3.3	4.5±3.4	0.10	
**Satisfied with health status**	0-10				0.97
**Run-in**		6.8±2.0	7.0±1.8	0.65	
**10-week**		6.9±2.2	6.6±2.4	0.63	
**Number of days doing vigorous PA**					0.88
**Run-in**		1.7±1.2	2.2±1.6	0.16	
**10-week**		2.0±1.8	1.7±1.7	0.48	
**Minutes per day doing vigorous PA**					0.52
**Run-in**		60.2±61.3	49.8±32.9	0.43	
**10-week**		43.8±48.8	37.2±36.4	0.55	
**Number of days doing moderate PA**					0.23
**Run-in**		3.2±2.1	3.1±2.1	0.75	
**10-week**		3.0±2.4	2.2±2.2	0.16	
**Minutes per day doing moderate PA**					0.18
**Run-in**		44.8±42.6	58.0±61.5	0.35	
**10-week**		42.9±39.4	58.2±81.9	0.36	
**Number of days walking**					0.52
**Run-in**		5.7±1.6	5.7±1.9	0.98	
**10-week**		6.1±1.33	5.7±2.0	0.28	
**Minutes per day walking**					0.41
**Run-in**		43.1±32.1	42.9±34.9	0.99	
**10-week**		54.6±49.4	57.1±61.2	0.86	
**Hours per day sitting**					0.32
**Run-in**		8.8±4.7	9.4±5.6	0.66	
**10-week**		7.7±2.5	9.4±9.5	0.34	

**Barriers to Being Active**					0.41
**Total score**	0-63(63 being greatest barrier)				
**Run-in**		19.1±8.7	20.9±10.3	0.45	
**10-week**		18.1±9.5	20.3±10.6	0.39	
**Lack of time**	0-9				0.51
**Run-in**		4.1±2.2	4.3±2.7	0.71	
**10-week**		4.2±2.5	4.0±2.8	0.83	
**Social influence**	0-9				0.55
**Run-in**		2.4±1.6	2.9±2.2	0.34	
**10-week**		2.2±1.6	2.6±2.0	0.39	
**Lack of energy**	0-9				0.24
**Run-in**		4.5±2.1	4.7±2.4	0.73	
**10-week**		4.3±2.3	4.4±2.4	0.91	
**Lack of willpower**	0-9				0.99
**Run-in**		4.3±2.6	4.4±2.7	0.93	
**10-week**		3.7±2.3	4.8±2.9	0.11	
**Fear of injury**	0-9				0.74
**Run-in**		0.7±1.2	0.6±0.9	0.74	
**10-week**		0.7±1.3	0.6±1.1	0.85	
**Lack of skill**	0-9				0.40
**Run-in**		1.3±1.8	1.2±1.6	0.96	
**10-week**		0.9±1.2	1.1±1.4	0.49	
**Lack of resources**	0-9				0.35
**Run-in**		1.9±1.7	2.8±1.6	**0.03**	
**10-week**		2.2±1.8	2.8±1.6	0.17	
